# Treatment of Brachial Plexitis: A Complication of Brachial Plexus Regional Nerve Block

**DOI:** 10.7759/cureus.30105

**Published:** 2022-10-09

**Authors:** Mohammad A Alkheder, Jad Said, Daniel J VanZweden

**Affiliations:** 1 Department of Internal Medicine, Beaumont Hospital, Dearborn, USA; 2 Medical School, Wayne State University School of Medicine, Dearborn, USA

**Keywords:** mssa bacteremia, staph aureus, brachial plexus, us guided abscess drainage, mssa abscess, brachial plexitis, brachial plexus blockage

## Abstract

Brachial plexitis is a rare condition characterized by inflammation within the brachial plexus presenting with acute shoulder pain, with motor and sensory deficits of the upper extremity. This case involves a 56-year-old female presenting with brachial plexitis after undergoing rotator cuff repair with a regional nerve block to the right brachial plexus. The diagnosis was made on her clinical presentation of fever with swelling and pain over her right shoulder and imaging showing inflammation and abscess formation with the soft tissue. The case was managed with antibiotic therapy and ultrasound drainage of the abscess.

Although this case is a rare occurrence, the likely etiology was secondary to peripheral nerve catheter placement for anesthesia, as well as her probable immunocompromised state with underlying uncontrolled diabetes mellitus. Surgical intervention was avoided because of the high probability of intraoperative complications due to the abscess’s location and thus management opted for less invasive measures.

Therefore, brachial plexitis with abscess formation is a complication of plexus nerve block, can present without any neurological deficit and is best managed conservatively without surgery.

## Introduction

It is very rare to have an abscess form in the base of the neck close to the brachial plexus causing brachial plexitis as a complication of a peripheral nerve block. Early detection, drainage, antibiotics, and conservative management, in general, are crucial in avoiding nerve damage and further neurological complications. In this case report, we discuss brachial plexitis incidence in a middle-aged woman that was treated conservatively and who recovered with no neurological complications despite the challenges imposed by her uncontrolled diabetes mellitus.

## Case presentation

The patient is a 56-year-old woman with a past medical history of hypertension, insulin-dependent diabetes mellitus, and a 30-pack-year history of tobacco use, who presented to the emergency department with a chief complaint of swelling at the right base of her neck after right shoulder arthroscopy with local nerve block 38 days prior to presentation. The patient stated that the mass was painful, and the swelling had been worsening since being noticed in the past week. The patient denied any fever or chills, difficulty breathing, eating, or swallowing, or any numbness or tingling in the neck, arm, or hand. She had no other complaints, and no pertinent family or social history.

A CT scan with contrast performed by the orthopedist two days prior to presentation revealed loculated fluid collection in the right lateral neck measuring 3.3 x 1.9 x 2.0 cm with surrounding fat inflammatory changes. The radiologist's report included a differential diagnosis of abscess, necrotic lymph node, or complex cystic mass, causing brachial plexitis. The patient was subsequently admitted the same day for workup and drainage of fluid.

Investigations

**Figure 1:**
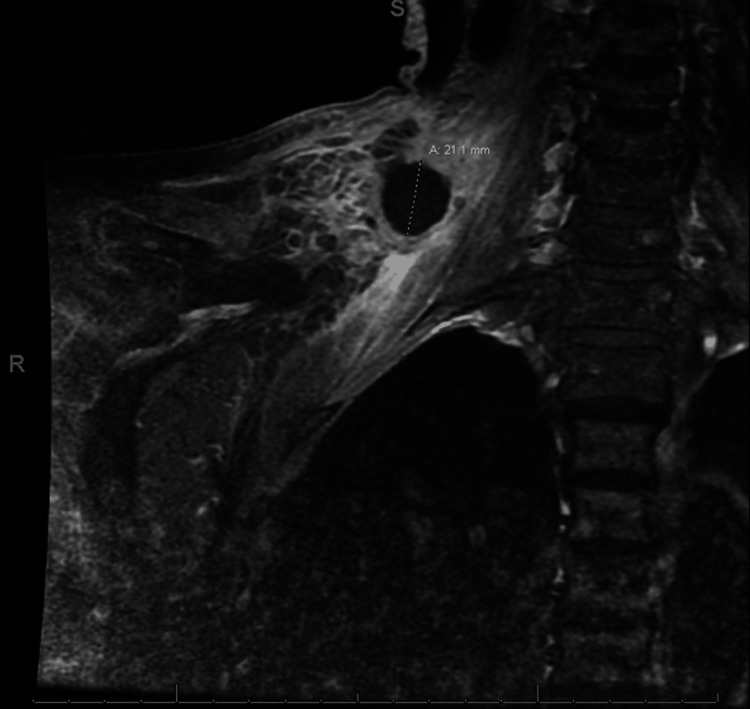
Diffuse soft tissue edema and enhancement in the right paraspinal and scalene musculature, with associated edema and enhancement along the right brachial plexus from proximal roots of the plexus to the mid clavicle.

An MRI (with and without gadolinium contrast) the following day after admission showed diffuse deep soft tissue edema and enhancement in the right neck involving the right paraspinal and scalene musculature, with associated edema and enhancement along the right brachial plexus from proximal roots of the plexus to the mid clavicle. Fluid collection in the region of edema measured 3.0 x 1.9 x 2.1 cm and was 5.2 cm deep from the skin surface (Figure 1). Ultrasound-guided drainage was done by interventional radiology, removing 10cc of white fluid. Laboratory analysis revealed pan-susceptible Staphylococcus aureus.

**Figure 2:**
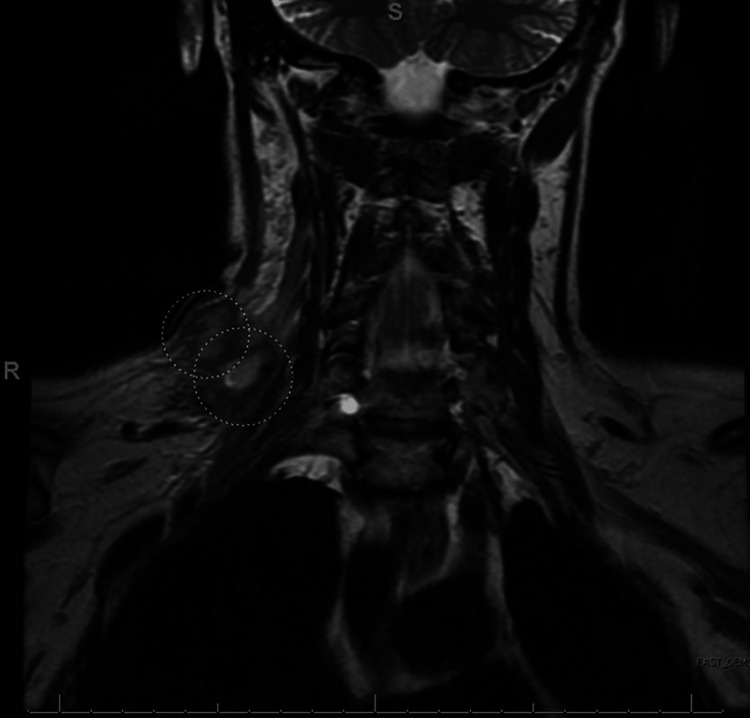
Decrease in abscess size to 2.29 x 1.41 cm compared to prior imaging with response to antibiotic therapy.

A follow-up MRI was performed three days later showing an unchanged abscess with some enhancement of the brachial plexus compatible with a degree of brachial plexitis. Another ultrasound-guided drainage removed 5 CCs of red fluid, and the culture grew the same organism. Three days later the final MRI showed decreased abscess size of 2.29 x 1.41 cm (Figure 2). The patient stated she had no pain, and the physical exam revealed a slightly indurated mass; she was subsequently discharged from the hospital on IV cefazolin with instructions to follow up with her primary care physician.

Treatment

When the patient presented to the emergency department, her physician was initially worried about purulent cellulitis caused by Staphylococcus aureus, partially because of the lesion being too close to the skin. She was started on oral clindamycin 300 mg every six hours for three days until her ultrasound-guided drainage could be completed. During that time, she failed to improve.

On the day of the patient’s first ultrasound-guided drainage, she initially received one intravenous dose of vancomycin 1000 mg, followed by piperacillin/tazobactam 3.375 grams every 6 hours for 2 days, and clindamycin 600 mg every 8 hours for 6 days as empiric coverage for Pseudomonas aeruginosa, Methicillin-resistant Staphylococcus aureus, and oropharyngeal gram-negative bacteria.

The following day, after laboratory culture returned revealing pan-susceptible Staphylococcus aureus, she began IV cefazolin 2 grams Q8 hours. She was discharged eight days after starting the cefazolin and continued to receive cefazolin through a peripherally inserted central catheter (PICC) for 16 more days after discharge with the help of skilled nursing visits at home.

Outcome and follow-up

The patient was re-admitted for PICC line infection 16 days later. Wound cultures returned positive for Pseudomonas aeruginosa; she was placed on cefepime. Her PICC line was removed, a midline was placed the following day, and she was subsequently discharged on doxycycline (100 mg BID for 10 days) and levofloxacin (500 mg for 10 days). The patient made a complete and full recovery with no deficits or further complications.

## Discussion

Discussion

Brachial plexitis presents with acute shoulder pain associated with motor and sensory deficits of the upper extremity. It is a rare occurrence with a disease incidence of 1.64 cases per 100,000 person-year, commonly more in males, with a peak onset between the third and seventh decade [1]. The case describes brachial plexitis secondary to peripheral nerve catheter (PNC) placement complicated by abscess formation. Although abscess formation is rare with a rate of 0-0.9%, multiple factors are likely contributing to the etiology such as high rates of bacterial colonization ranging from 7.5%-57%, with axillary PNC having the highest rates. Past medical history of uncontrolled diabetes mellitus causing an immunocompromised state may have also added to the likelihood of PNC complications [2].

Multiplanar multi-sequence MRI with and without gadolinium detected diffuse deep soft tissue edema surrounding the abscess involving the right paraspinal musculature associated with enhancement along the course of the right brachial plexus from the proximal roots to the anatomic level of the mid-clavicle. This indicates inflammation involving the brachial plexus, which may cause a neurological deficit in the right upper limb with associated edema. Around 95% of cases present with shoulder pain, combined with simultaneous or delayed weakness [3]. Since neural function was not affected in this case, and the limitation of movement of the right upper limb was secondary to pain and not weakness, brachial plexitis was likely mild in severity.

Management of the brachial abscess involving a surgical intervention was avoided due to its difficult location near the proximal right brachial plexus, and possible intraoperative phrenic nerve injury by traction or transection could likely result in diaphragmatic injury [4]. Instead, interventional radiology performed ultrasound-guided drainage over two sessions removing all accumulated fluid. The patient continued to be febrile and was started on broad-spectrum antibiotics with clindamycin, vancomycin, and piperacillin-tazobactam. Fluid cultures were taken which subsequently grew methicillin-sensitive Staphylococcus aureus (MSSA), and antibiotics were switched to clindamycin for a total of 10 days, and IV cefazolin for four weeks. Her IV regimen was continued as an outpatient after the placement of a peripherally inserted central catheter (PICC) line.

The severe neck, shoulder, and right arm pain present since admission was managed with opioid analgesics, non-opioid analgesics, and non-steroidal anti-inflammatory drugs (NSAIDs). Symptoms were alleviated, likely targeting the surrounding edema and the pain that was reportedly limiting upper limb movement [5]. Follow-up as an outpatient and concomitant physical therapy subsequently resulted in full recovery.

## Conclusions

Regional nerve block might contribute to abscess formation, especially in a patient with uncontrolled diabetes mellitus. In this case, the abscess was close to the brachial plexus and eventually would lead to brachial plexitis, which if not treated appropriately in a timely manner could aggregate further to neurological complications. Ultrasound-guided drainage of the abscess would be preferred over surgical incison and drainage because of the risky location close to the plexus. Early start of antibiotics and strict diabetes control are essential for brachial plexitis recovery with no lasting complications.
